# Diverse stimuli induce piloerection and yield varied autonomic responses in humans

**DOI:** 10.1242/bio.060205

**Published:** 2024-08-08

**Authors:** Jonathon McPhetres

**Affiliations:** Durham University

**Keywords:** A-delta, Affect, Arousal, Autonomic, C-tactile, Evolution, Emotion, Goosebumps, Human, Integumentary system, Piloerection, Sympathetic, Tactile stimulation, Thermoregulation

## Abstract

This research provides an in-depth exploration into the triggers and corresponding autonomic responses of piloerection, a phenomenon prevalent across various species. In non-human species, piloerection occurs in reaction to a variety of environmental changes, including social interactions and temperature shifts. However, its understanding in humans has been confined to emotional contexts. This is problematic because it reflects solely upon subjective experience rather than an objective response to the environment. Further, given our shared evolutionary paths, piloerection should function similarly in humans and other animals. I observed 1198 piloerection episodes from eight participants while simultaneously recording multiple autonomic and body temperature indices, finding that piloerection in humans can be elicited by thermal, tactile, and audio-visual stimuli with equal effectiveness. The data also revealed variations in cardiac reactivity measures: audio-visual piloerection was associated with greater sympathetic arousal, while tactile piloerection was linked to greater parasympathetic arousal. Despite prevailing notions of piloerection as a vestigial response in humans, it does respond to decreases in skin temperature and is associated with a rise in skin temperature during episodes. This research underscores that piloerection in humans is not purely vestigial, nor is it solely an affective response to emotional stimuli. Rather, it is best understood as a reflexive response to environmental changes, suggesting a shared functional similarity with other species.

## Teaser

**Summary:** This study explores the multifaceted triggers of human piloerection, revealing complex autonomic and thermoregulatory responses, and challenging the view of it as a mere vestigial or emotional response.

## INTRODUCTION

The integumentary system is a highly sensitive organ involved in many aspects of biological functioning, including in perceiving and responding to environmental changes. One of these responses is piloerection: the contraction of the arrector pili muscle, causing the hair to stand erect and forming visible bumps on the skin.

Evident across a diverse range of non-human species, piloerection can be triggered by a variety of stimuli. Avian species may ruffle feathers as part of courtship rituals (Dakin et al., 2016), to intimidate rivals (Royer and Anderson, 2014), or in response to cold to retain heat (Tansey and Johnson, 2015). Among mammals, the phenomenon is noticeable during threats (Pruetz et al., 2022 preprint; Heesen et al., 2022; Muller and Wrangham, 2004) and mating displays (Converse et al., 1995), and also as a thermoregulation mechanism (Chaplin et al., 2013). This physiological process, therefore, plays a role in social interaction, signalling, and in maintaining the organism's thermal equilibrium.

While piloerection serves clear functions in non-human animals, the same cannot be said with certainty when it comes to humans. In fact, very little is known about human piloerection. Research in biology and psychology diverges in focus, yet neither discipline has sufficiently addressed what precisely triggers piloerection or its underlying autonomic responses in humans. Biological studies often emphasise the molecular and physiological mechanisms behind piloerection, missing insights into its practical implications or triggers in everyday human experiences. On the other hand, psychological research has gravitated towards the emotional or subjective experiences associated with piloerection, such as the sensation of ‘the chills’, without robustly investigating the physicality or the diversity of stimuli that could induce this response. To restate, no research to date has demonstrated the various types of stimuli that can induce piloerection in humans and the resulting autonomic responses.

Further, despite frequent references to piloerection in relation to emotions (McPhetres and Zickfeld, 2022) or as a presumed vestigial reflex (Werth, 2014; Heathers et al., 2018; DeWitt, 2008), there is a surprising lack of empirical research dedicated to verifying these assumptions. Critically, no research has demonstrated the thermoregulatory effects of piloerection in humans. This gap points to a significant oversight: piloerection is often discussed in terms of its observable outcomes – whether emotional or physiological – without a foundational understanding of its causative factors or the spectrum of autonomic responses it may encompass.

This discrepancy between human and animal models brings forth an intriguing question: given our shared evolutionary paths, is it not plausible that piloerection functions similarly in humans as in other animals? Are we oversimplifying the human experience of piloerection by attributing it mostly to emotional experiences, overlooking the fact that humans can likely experience piloerection in response to a range of stimuli?

Adding to this complexity, various stimuli have distinct neural pathways. For instance, tactile stimuli are detected by afferent c-tactile fibres, and thermal information is passed from thermoreceptors along afferent Aδ fibres (Glatte et al., 2019). In contrast, audio-visual stimuli – be it a moving symphony or an unexpected sound and rustling in the treeline – utilise optical and auditory routes, and these engage a different set of neural processes. Yet, these diverse routes all converge to activate the same efferent sympathetic fibres (Donadio et al., 2019), which innervate the arrector pili muscles. This leads to a critical question: given that multiple paths converge to initiate the same final response, is piloerection a singular physiological reaction, or do the underlying autonomic responses reflect the diverse stimuli? At the same time, we ask another question: is piloerection a purely vestigial reflex, or does it serve a functional role in thermoregulation that is not contingent on the stimulus that triggers it?

## RESULTS

In total, 1198 piloerection episodes were recorded across the three blocks. Tactile, thermal, and audio-visual stimuli were equally likely to cause piloerection (*R*^2^=0.03, *P*=0.180; see Table S1) – that is, all participants experienced piloerection in response to each type of stimuli, with the exception that one participant did not experience piloerection in the cold room. However, there was some heterogeneity in the number of piloerection episodes resulting from specific tasks within each block (η_p_^2^=0.10, *P*=0.001), likely due to variation in task length. For example, the cold room exposure lasted 20 min while the videos and other tactile tasks ranged from 1-3 min (see Table S2). Thus, these results demonstrate that multiple stimuli routes yield a singular physiological response in human subjects.

### Audio-visual piloerection reflects sympathetic arousal

To examine autonomic arousal, cardiovascular metrics were calculated for the 10 s around each piloerection event. A reactivity score for each metric was then computed by subtracting the baseline score from the piloerection epoch, and a linear mixed-effects model (with random intercepts for subject ID) compared the three blocks on each reactivity score. A summary of key metrics of sympathetic and parasympathetic arousal indices is presented in Table 1, below. Full metrics are reported in Table S3.

**Table 1.**
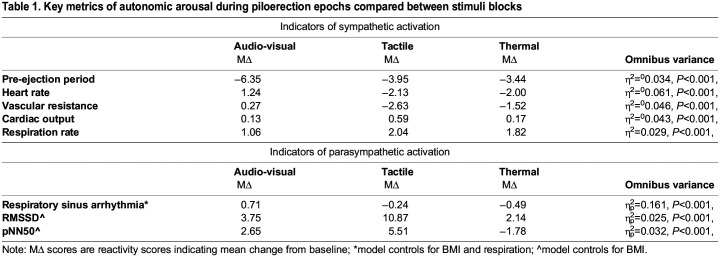
Key metrics of autonomic arousal during piloerection epochs compared between stimuli blocks

Audio-visual piloerection showed a greater decrease in pre-ejection period (a key indicator of sympathetic arousal), coupled with an increase in heart rate, vascular resistance, and cardiac output. Together, this pattern of results indicates that piloerection elicited by audio-visual stimuli is associated with a stronger sympathetic arousal pattern compared to tactile and thermal piloerection. In contrast, tactile and thermal piloerection showed decreases in heart rate and vascular resistance.

At the same time, tactile stimuli showed stronger evidence of parasympathetic arousal as indicated by a large increase in two out of three metrics of heart-rate variability; the root mean square of successive differences (RMSSD) and the proportion of normal inter-beat intervals greater than 50 ms (pNN50). A third metric, respiratory sinus arrhythmia, showed results in the opposite direction, but respiratory sinus arrhythmia (RSA) and pre-ejection period can frequently correlate positively (Weissman and Mendes, 2021).

### Tactile piloerection reflects dermatome innervation

Recent studies have demonstrated that piloerection occurs consistently across the body (McPhetres et al., 2024b), contrary to prevalent beliefs that it is more common on the forearm. I examined this here, as well, by computing correlations between the number of piloerection episodes at each anatomical location. Findings indicated that tactile piloerection showed notably lower correlations compared to those caused by audio-visual and thermal stimuli (see Table 2), which I attribute to the unique innervation patterns within each dermatome.

**Table 2.**
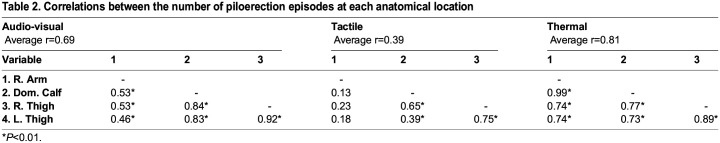
Correlations between the number of piloerection episodes at each anatomical location

Specifically, the largest correlation under tactile stimulation appeared between the right and left thigh (*r=*0.75), likely due to the shared innervation by the L3 spinal nerve. Similarly, the right calf, served by the L4 nerve, demonstrated a stronger correlation with the right thigh (*r*=0.65) compared to the left (*r*=0.39). Notably, the upper arm, innervated by the C5 spinal nerve, showed weak correlations with lower extremity piloerection. In contrast, audio-visual and thermal stimuli showed much stronger correlations across the anatomical locations. This is potentially due to the stimuli input route: audio-visual stimuli were processed through optical and auditory channels, while thermal stimuli impacted the entire body, as in the case of exposure to a cold room. Thus, these findings suggest that tactile piloerection is anatomically specific, triggered according to the location of sensory stimulation, whereas audio-visual and thermal piloerection generate a more systemic response.

### Piloerection responds to, and affects, skin temperature

Contrary to the conception of piloerection as a vestigial trait, the data also showed that piloerection is associated with changes in skin temperature. To visualise this, the data were processed in several steps.

First, 15 s of temperature data prior to and following the piloerection peak were selected. Because piloerection events across the body could occur within a few seconds of each other, each hypothesised to affect skin temperature, piloerection events were excluded if another event took place within that 15 s window. This allows for only prototypical events to be analysed and depicted.

Next, the rolling average of piloerection events was calculated for each person using a 10-s rolling average function. A 10-s window was chosen because piloerection can last for 10 s or more (McPhetres et al., 2024a). Additionally, there could be multiple piloerection events within 1 s of each other and these would, in all likelihood, constitute the ‘same’ event. Further, each person had four cameras, so the rolling average function quantifies the number of piloerection events within that time frame essentially yielding an intensity score. Therefore, a rolling average function helps to both smooth out the data and aggregate regions of time where piloerection was experienced. The time window of the rolling average does not greatly affect the data, but it will make the resulting graphs easier to interpret.

**Fig. 1.**
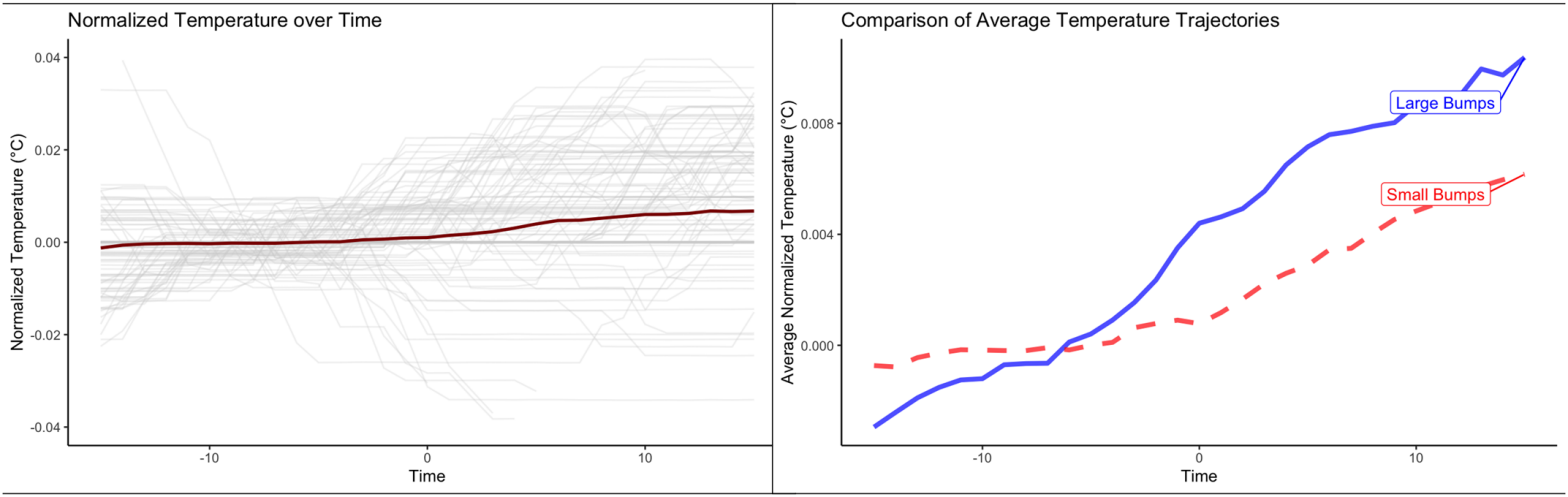
**Piloerection is preceded by a decrease in skin temperature and followed by an increase in skin temperature during the piloerection epoch.** Note: Time 0 indicates the peak of the piloerection event.

Fig. 1 shows that piloerection is preceded by a decrease in skin temperature, followed by an increase in temperature during the resulting piloerection epoch. This is evident in several analyses.

Cross-lagged correlations (see Fig. S1) indicated that the correlation between skin temperature was strongest about 8-10 s prior to the peak of the piloerection episode. A linear regression confirms that a decrease in skin temperature at Lag −8 predicts a greater intensity of piloerection (B=-0.98, SE=0.28, *P<*0.001). Changes in oral temperature at Lag 8 were not related to piloerection (B<0.01, SE=0.02, *P=*0.900). Because of this correlation, plus the fact that the temperature change during piloerection epochs was significant only for skin temperature, the next analyses focused only on skin temperature.

Then, to test the hypothesis that more intense episodes of piloerection would result in greater changes to skin temperature, the intensity of the piloerection event was considered. Piloerection events were coded as ‘large’ (fully formed bumps symmetrical around the infundibulum) or ‘small’ (twitches or bumps in-between the infundibulum that dissipated before developing into large bumps). A recent study provides examples of the varying intensities of piloerection (McPhetres et al., 2024a).

Next, detrended axillary temperature at this lag time was used to predict the intensity of piloerection events (calculated as the rolling average of piloerection across all body locations) in a linear-mixed effects regression model. Larger decreases in skin temperature predicted more intense piloerection events (B=-0.98, *P<*0.001); piloerection was not related to preceding oral temperature (B<0.01, SE=0.02, *P=*0.900).

Second, skin temperature change from baseline around each piloerection event was predicted with dummy codes for task type (audio-visual versus tactile versus thermal) in a mixed-effects model. Axillary temperature was higher during piloerection epochs, and this increase in temperature varied according to stimuli type (η^2^=0.195, *P*<0.001). Relative to baseline, skin temperature increased about 0.18°C (95% CI: −0.02, 0.39) during audio-visual piloerection, 0.29°C (95% CI: 0.09, 0.50) during tactile piloerection, and 0.36°C (95% CI: 0.15, 0.57) during thermal piloerection. Core (sublingual) temperature, however, did not respond to stimuli type (η^2^<0.01, *P*=0.108). Fig. S2 displays detailed temperature trajectories according to piloerection intensity.

Finally, to test the hypothesis that more intense piloerection episodes would be associated with a greater change in skin temperature, a linear mixed-effects model predicted skin temperature with Time and piloerection size (see also Table S5). Results indicated that small piloerection events were associated with a continually smaller increase in temperature compared to large piloerection events (B=-0.0002, *P<*0.001). For example, at 15 s after the peak of piloerection, skin temperature during ‘large’ piloerection events was about 0.004°C warmer than ‘small’ piloerection events (Z=6.33, *P<*0.001, Cohen's d=0.62).

## DISCUSSION

Discussion focused on animals treats piloerection as a response mechanism to a variety of stimuli, as a social signalling mechanism, and for thermoregulation, whereas humans are believed to experience this almost exclusively as an emotional response (McPhetres and Zickfeld, 2022). However, the findings from this study challenge this perspective. These results show that piloerection in humans can be triggered by a diverse range of stimuli, including tactile, thermal, and audio-visual cues. These stimuli also had varying autonomic effects, including cardiovascular and thermal changes. These insights hold significant implications across the life sciences, as they underscore the necessity to review the dichotomous models traditionally used to interpret piloerection in humans versus animals.

### Stimuli

One salient implication of these findings is that human piloerection can be induced by a similar spectrum of stimuli as those affecting animals. While this may seem to be an obvious claim, it has yet to be documented in the wider literature. In fact, psychological literature almost exclusively treats piloerection as an emotional response, yet the current results clearly demonstrate that piloerection can occur in response to non-emotional triggers. Although the functional role of piloerection in humans has become less pronounced – we no longer rely on hair erection as an indicator of impending threats, we have clothes for thermoregulation, etc. – its physiological and sensory precursors have been conserved. Environmental changes bear a wealth of information and can spark significant physiological responses. These changes, whether in the form of acoustic and visual cues or temperature and tactile sensations, engender human reactions much as they would in animals. Thus, a gentle touch or a shift in temperature can induce piloerection, as could a sudden auditory or visual cue. The audio-visual stimuli employed in this study have much in common with the sensory experiences animals encounter in their natural environments. Moving music or frightening videos provoke in us physiological responses, akin to the reactions animals may display when confronted with distress signals from peers (Heesen et al., 2022) or potential threats (Pruetz et al., 2022 preprint). This underscores a more comprehensive role of piloerection beyond subjective emotional responses in humans. However, much more research is needed to identify what qualities of such stimuli are specifically responsible for prompting the piloerection response.

### Thermoregulation

This study illustrates that these varied stimuli were consistently associated with changes in skin temperature. While core body temperature (as measured sublingually) remained unaltered, skin temperature demonstrated notable fluctuations prior to and during piloerection events. This highlights that, despite previous characterisation of piloerection as a vestigial response in humans (Heathers et al., 2018; Torkamani et al., 2017), the present findings offer evidence that it retains elements of its functional characteristics. Notably, piloerection influenced skin temperature in the axillary area regardless of the stimulation method or region. For example, piloerection may have been witnessed on the arm, but the temperature change was recorded in the axilla. This universal physiological response to diverse stimuli supports the idea that piloerection retains a fundamental role in the body's thermoregulatory processes. Moreover, while sublingual core temperature remained consistent, reinforcing the body's tendency toward internal thermal regulation, the localised skin temperature changes during the piloerection episodes indicate a nuanced interaction between surface-level thermoregulatory mechanisms and environmental factors.

The elicitation methods also shed light on the nuanced nature of the thermoregulatory response. Cold ambient temperatures typically triggered a more systemic piloerection response, suggestive of an overall body thermoregulatory effort, whereas tactile stimuli often led to a localised effect, suggestive of dermatome-specific activation. Such variations in piloerective responses underscore a complex system that might still serve a thermoregulatory purpose, albeit in a more targeted manner than previously understood.

However, it is important to consider that there are many other factors involved in thermoregulation, such as vasoconstriction/dilation, which were not considered here. Thus, these results do not claim to show that piloerection is solely or directly responsible for skin temperature changes because it is only one part of a systemic response. Still, these findings contribute to a growing body of evidence that human piloerection, far from being purely vestigial, may play a nuanced and functionally significant role in skin thermoregulation, paralleling some aspects of animal physiology.

### Psychological understanding

The implications of these findings also have a far-reaching impact on the field of psychology. The primary interpretation of piloerection within psychological literature has been tied to emotional states. This study, however, shows that piloerection should be reframed as a response to changes in environmental stimuli, shifting the focus away from its association with psychological experiences. Within this psychological literature, piloerection is commonly coupled with emotions typically linked to parasympathetic activity (for example, see a discussion around the emotion of awe, Quesnel and Riecke, 2018; McPhetres and Shtulman, 2021; Keltner and Haidt, 2003). However, it is well-established that the arrector pili muscles are innervated by the sympathetic nervous system (Glatte et al., 2019; Donadio et al., 2019). Nevertheless, this study observed nuanced cardiovascular responses in conjunction with piloerection, contingent on the nature of the eliciting stimuli. For example, while audio-visual stimuli corresponded with elevated sympathetic cardiovascular activity, tactile stimuli led to heightened parasympathetic responses. This is consistent with past literature, which has found light pleasurable tactile stimulation to be experienced as pleasurable in past research (Pawling et al., 2017). These findings challenge the emotion-centric view of piloerection, which erroneously associates it with parasympathetic activation, and calls for a more comprehensive understanding of this physiological phenomenon in humans.

### Further considerations

Several additional factors warrant consideration in interpreting the findings of this study. Notably, the observation of piloerection was confined to specific areas and did not encompass the entire body, but this is a necessary limitation because participants are already wearing physiological equipment all over their body and additional cameras would be burdensome. Skin temperature measurements were similarly limited to single locations, which precludes us from fully understanding the potential variations across the body's surface. In this sense, I was also limited by technological possibilities and so chose to observe skin temperature in a region that would be protected from ambient temperature changes. Hair density, which I did not measure, could also influence the thermoregulatory effects observed; individuals with varying hair density or those with shaved skin might exhibit stronger or weaker temperature responses. While these factors may provide additional nuance to the results obtained here, I do not expect that they would undermine or substantially alter the findings presented.

Another factor is the sample size. The study design facilitated a substantial number of within-person observations, amounting to 1198 instances, thus providing statistical power to detect even negligible effect sizes. There are several strengths to this approach, including the ability to smooth out intrapersonal variations, control for individual differences, and increase statistical power. However, the matter of generalisability extends beyond mere numbers. The elicitation methods employed to induce piloerection were modelled after real-life scenarios, bolstering the interpretation that piloerection can be elicited in many ways.

A key strength of this research lies in the demographic breadth of the data, encompassing a wide range of ages and body mass indices (BMIs). This suggests that the piloerection responses observed are consistent across different ages and body types. While the actual physiological efficacy of piloerection and the participants' awareness of these changes might be modulated by age – indeed, a recent study indicated a general lack of awareness about the occurrence of piloerection (McPhetres et al., 2024b) – these factors would not substantially affect the observed physiological responses.

Looking ahead, future research could further delineate the relationship between piloerection and thermoregulation by examining how different piloerection intensities affect temperature regulation. It would also be informative to explore if certain stimuli or circumstances are more likely to elicit varying levels of piloerection intensity. Such inquiries would deepen our understanding of this complex physiological phenomenon and its implications across the lifespan.

### Conclusions

In conclusion, the present study presents a transformative perspective on the occurrence and understanding of piloerection in humans. Contrary to the predominant view that assigns an almost exclusive emotional basis to this phenomenon, these findings suggest that piloerection is a nuanced physiological response to a variety of environmental stimuli. This aligns more closely with the multifaceted stimuli-response nature of piloerection observed in animal models. Furthermore, this study demonstrates the need for a shift in focus from emotional to environmental stimuli within psychological literature on piloerection. The nuanced cardiovascular and thermoregulatory responses observed here, contingent on the nature of the stimuli, call for a more comprehensive and inclusive study of piloerection. This research redefines piloerection as a complex and dynamic physiological response rather than a vestigial one, opening up new paths for future investigations in both physiological and psychological domains.

## MATERIALS AND METHODS

### Participants

The study was designed to maximise the number of piloerection events observed within each participant. This research involved eight right-handed participants (k=1198 piloerection events), five females and three males, aged between 20 to 63 years old (M=28.63, SD=15.39) with BMIs ranging from 15.5 to 34.2 (M=28.6, SD=15.4), drawn from the university and surrounding community. Participants were healthy, with no illnesses, injuries or medications.

### Overview

Data collection took place between March and April of 2023. All sessions took place at 10am and each laboratory session took around 3 h. In short, participants arrived at a laboratory wearing shorts and a t-shirt. They completed informed consent, had their height and weight measured and were connected to physiological equipment.

All stimuli tasks were pseudo-randomised into blocks such that, for example, all feather applications took place together in a block, and all video displays took place together in a block. Participants remained seated in a chair for the entire segment. After each task, participants were asked to self-report their emotional valence (on a scale from extremely negative to extremely positive) and ‘warmness’ (on a scale from ‘freezing’ to ‘too hot’). These data are not relevant to the current report and will be described in a forthcoming article.

### Physiological recording

Physiological data was recorded through a BioPac MP160 (BioPac Systems, UK) using Acqknowledge v 5.0. ECG data was recorded through the Bionomadix RSPEC-R using a lead II configuration with Ag/AgCl spot electrodes (EL-503). Impedance cardiography was recorded through the Bionomadix NICO-R using paired Ag/AgCl spot electrodes (El-500) in an eight-spot configuration, and the distance between the electrodes was measured. Blood pressure (BP) was recorded through a CNAP continuous blood pressure monitor (CN Systems, Austria) using the CNAP finger sensor. Axillary skin temperature was measured using the skin temperature thermistor (TSD202B, BioPac Systems, UK) placed on the lower axilla against the ribs and was recorded through the SKT2-R Bionomadix module. Core body temperature was recorded via a sublingual thermometer (TSD202F, BioPac Systems, UK) inside a plastic sheath, recorded via the SKT100c (BioPac Systems, UK).

Physiological data were recorded using Acqknowledge (v 5.0) and were pre-processed and analysed using MindWare programs HRV (v 3.2.9), BPV (v. 3.2.5), IMP (v. 3.2.10), and BSA (3.2.9). Specifically, data were viewed in 10-s segments centred around each piloerection event during stimulation tasks and in 1-min segments for other tasks (e.g. videos, cold room); artefacts were identified visually and corrected, and SV was calculated using the Kubicek method. Respiration was calculated from impedance by applying a 5 hz filter to the dZ/dt impedance values via MindWare. Computed values were exported and combined in R (v. 4.3.0).

### Piloerection

Piloerection events were observed through four high-definition cameras positioned to capture the dominant upper dorsolateral arm, dominant dorsal calf, and both left and right anterolateral thighs. Cameras were synced to Acqknowledge via an LED light (OUT103, BioPac Systems, UK) and viewed later in video coding software (BORIS v. 8 Friard and Gamba, 2016) second-by-second to identify the peak of each piloerection event. In short, the video was viewed at increased speed using the ‘slider’ function to move backwards and forwards over successive ∼20 s segments until piloerection was noticed. Piloerection was coded in a binary measure of intensity: either ‘large’ fully formed, obvious bumps symmetrical around the infundibulum, or ‘small’ less intense episodes, characterised by small twitches, which did not form into fully formed large bumps. A description of the morphology of piloerection is not the goal of the current research, but examples of the varying intensities of piloerection can be seen elsewhere (McPhetres et al., 2024a).

### Procedure

Participants were seated in a padded rolling chair while physiological equipment was placed. The chair did not rotate, or raise/lower, and the wheels were locked to prevent participant movement from interrupting recording. Participants were wheeled to and from each room, so they remained seated for the entire experiment. The participants underwent an acclimation period and the final minute of this was used as the baseline reference period. After the baseline, they were exposed to a series of stimuli blocks to induce piloerection. The blocks were pseudo-randomised, and the entire process spanned approximately 3 h. For tactile stimulation, the participants were in a room temperature (referred to here as the ‘warm room’) lab environment (M=20.28°C, SD=0.88°C). C-tactile afferents were stimulated by lightly brushing the participant's skin with a feather and a metal ‘tickler’ toy (Pawling et al., 2017), and administering two gentle puffs of air into the right ear using a rubber bulb aspirator (Hamalainen et al., 1985). These stimuli were applied to the area around each camera location and the nape of the neck.

Thermal stimulation targeted Aδ afferents by exposing participants to cold temperatures. This involved a 20-min stay in a separate air-conditioned ‘cold’ room (M=16.43°C, SD=0.41°C) while watching a neutral video, as well as direct skin contact with ice packs at each camera location and the nape of the neck, which was applied within the warm room and randomised in with the other stimuli.

In the audio-visual stimulation stage, participants were relocated to a private cubicle (within the warm-room) where they viewed a neutral video, followed by four piloerection-inducing videos (see Table S4) in randomised order. Two videos were chosen from previous research (McPhetres et al., 2024b) and are reported to cause piloerection in about 60% of participants (the most effective set of piloerection-inducing stimuli reported to-date). Two additional videos were chosen to present a variety of content, one from a previous article (Iriarte et al., 2024), and a fourth was selected by the author.

### Missing data

Due to an issue that prevented the CNAP from calibrating, BP data are missing from one participant entirely, during the cold room segment from one participant, and from the warm room for a third participant. This also means that metrics analysed at the same time (TPR, respiration) are not available for those segments. Additionally, one participant is missing BP values from one video segment due to movement artefacts. Physiologically implausible CO and TPR values (CO values less than 1 or TPR values greater than 50% of mean arterial pressure) were also removed. Finally, temperature data around six piloerection events were removed (three large and three small) due to a loss of signal at that time (see temperature analysis, below).

Physical stimuli (feather, tickler, and ice packs) were applied to the area around each camera. However, some locations were either inaccessible (due to the size/shape or position of participants) or were skipped because piloerection from the previous application persisted for too long and, instead, a warm paper towel or a hand was placed on the location to help dissipate the effect before moving on to the next location. As a result, the L thigh is missing two applications of the feather and one application of the tickler and ice pack; the arm is missing one application of the ice pack, and the calf is missing two applications of the feather, and three applications of the tickler and ice pack. No other issues were encountered.

### Ethics

This study received ethical approval from the Durham University Psychology Ethics Sub-Committee: PSYCH-2021-12-06T16_03_28-mqbg73. All subjects provided informed consent.

## Supplementary Material

10.1242/biolopen.060205_sup1 Supplementary information
