# Supplementary material for: Diverse stimuli induce piloerection and yield varied autonomic responses in humans
Source: Biol Open. 2024 Aug 8;13(8):bio060205. doi: 10.1242/bio.060205 (PMC11391818; doi:10.1242/bio.060205)
Supplement: Supplementary information [file biolopen-13-060205-s1.pdf]

Fig. S1. Cross-correlation function showing the correlation between the rolling average of piloerection and skin/oral temperature.

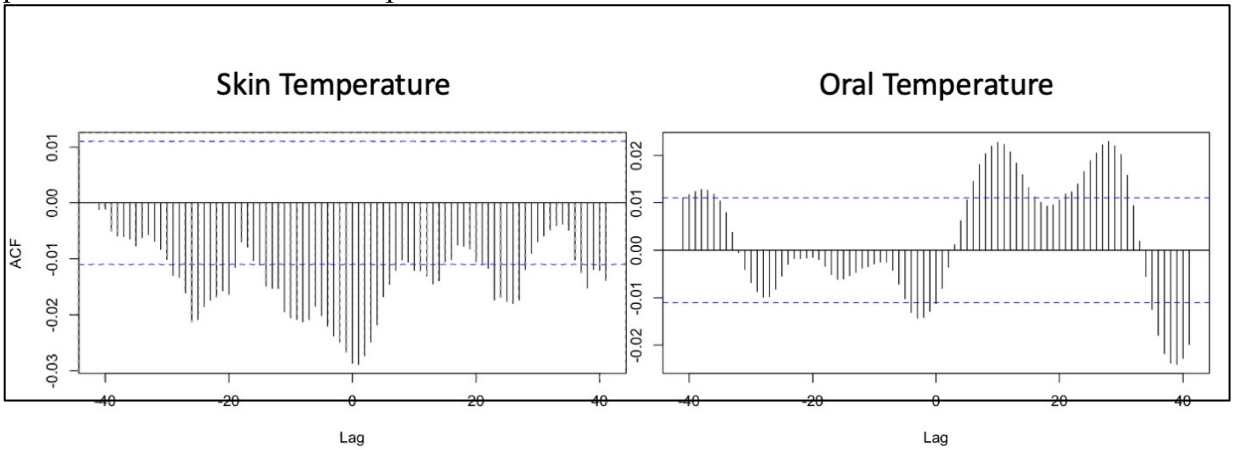

Note: Blue dashed lines indicate significance at p < .05; Because Lag 0 is the *peak* of the piloerection events and piloerection can last around 10 seconds, we would be looking for a temperature change that occurs around 10 seconds prior to each peak. We must also consider that the correlation function aggregates across all types of piloerection events—some would be shorter or longer than others—so there is likely some error in the correlation. This means that the peak occurring around lag 8 to 10 is much more plausible than the peak occurring at Lag 26 because Lag 8 would potentially be 2-3 seconds (on average) prior to the *beginning* of the piloerection event.

Fig. S2. Average skin temperature trajectories for large and small piloerection events.

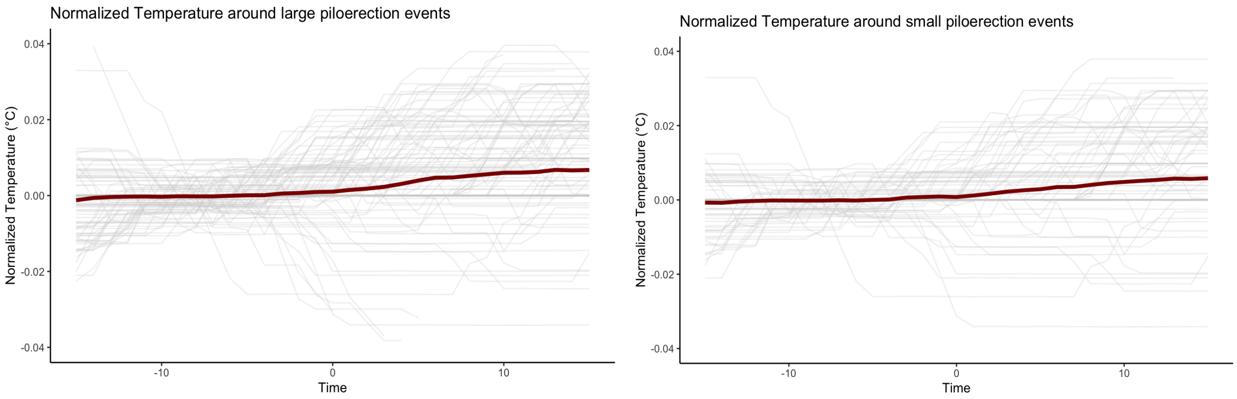

Note: Examination of these temperature trajectories reveals that the piloerection events (at Time 0) were preceded by a small decrease in temperature and followed by an increase in temperature *during* the piloerection event. The maximum decreases in temperature prior to piloerection was -.003°C for large piloerection and .001°C for small piloerection. The maximum increase in temperature during the piloerection event (after Time 0) was .01°C for large piloerection and .005°C for small piloerection.

Biology Open • Supplementary information

Table S1. Mixed-effects multiple regression comparing the likelihood of experiencing piloerection across three task blocks.

Task Block	B	SE	Z	P
Temperature	.56	.53	1.06	.290
Audio-visual	.94	.58	1.61	.110

Note: Tactile stimuli is the reference group.

Table S2. The number of piloerection events recorded at each anatomical location according to location of stimuli application.

**Air Puff**				
Stim Location	*R Arm*	*R Calf*	*L Thigh*	*R Thigh*
Ear	3 (27.3%)	3 (27.3%)	1 (9.1%)	4 (36.4%)
**Metal Tickler**				
R Arm	5 (9.8%)	5 (9.8%)	2 (3.9%)	4 (7.8%)
R Calf	2 (3.9%)	2 (3.9%)	1 (2.0%)	2 (3.9%)
Neck	3 (5.9%)	6 (11.8%)	3 (5.9%)	3 (5.9%)
L Thigh	2 (3.9%)	1 (2.0%)	1 (2.0%)	1 (2.0%)
R Thigh	1 (2.0%)	3 (5.9%)	3 (5.9%)	1 (2.0%)
**Feather**				
R Arm	3 (7.3%)	4 (9.8%)	3 (7.3%)	2 (4.9%)
R Calf	3 (7.3%)	1 (2.4%)		
Neck	3 (7.3%)	4 (9.8%)	2 (4.9%)	4 (9.8%)
L Thigh	1 (2.4%)			
R Thigh	3 (7.3%)	4 (9.8%)	2 (4.9%)	2 (4.9%)
**Ice Pack**				
R Arm	4 (6.1%)	2 (3.0%)	2 (3.0%)	2 (3.0%)
R Calf	2 (3.0%)	2 (3.0%)	1 (1.5%)	2 (3.0%)
Neck	6 (9.1%)	5 (7.6%)	5 (7.6%)	6 (9.1%)
L Thigh	4 (6.1%)	4 (6.1%)	2 (3.0%)	3 (4.5%)
R Thigh	3 (4.5%)	4 (6.1%)	3 (4.5%)	4 (6.1%)

Note: The variation in "effectiveness" of eliciting piloerection for each task is complicated by multiple factors, including the number of locations and total applications, as well as missing data. Thus, direct comparison of effectiveness of stimuli and the likelihood of eliciting piloerection, as well as the correspondence between application location and piloerection location are difficult. An overview of these stimuli suggests a few conclusions. The first is that the highest correspondence between application location and piloerection location was observed for the metal tickler (9.8% on the right arm and right calf). The second is that the neck appears to be the most effective stimuli location. The third is that no locations stand out as the most likely for piloerection to occur. Beyond these summary statistics, this study was not designed to test the effectiveness of varied stimuli—only to effectively elicit stimuli so that autonomic indices could be monitored.

Biology Open • Supplementary information

Table S3. Cardiovascular reactivity during piloerection epochs.

Index	Audio-visual M (SE)	Tactile M (SE)	Thermal M (SE)	p.value	η^2
PEP	-6.353215 (3.526587)	-3.954260 (3.558500)	-3.443167 (3.525160)	< .001	0.034
HR	1.236856 (1.544213)	-2.134072 (1.596386)	-2.002439 (1.541829)	< .001	0.061
TPR	0.2673985 (0.8531925)	-2.6276023 (0.9032103)	-1.5174465 (0.8488832)	< .001	0.046
SV	0.9048371 (1.639454)	9.2776466 (1.758542)	1.8497391 (1.632717)	< .001	0.072
CO	0.1249047 (0.1165757)	0.5858946 (0.1264443)	0.1668925 (0.1166174)	< .001	0.043
LVET	-1.2489126 (8.056316)	-2.1218485 (8.126347)	0.1302426 (8.053187)	.209	0.003
MAP	-2.234640 (3.988138)	-2.289768 (4.007482)	-2.769205 (3.982221)	.69	0.001
Resp. Rate	1.058474 (0.6133684)	2.044133 (0.6321852)	1.821816 (0.6153475)	< .001	0.029
Resp. Ampl.	-0.03813294 (0.1791821)	0.55509412 (0.2669990)	0.34041637 (0.1815441)	.084	0.007
RSA	0.7121632 (0.2863805)	-0.2413028 (0.2934904)	-0.4905804 (0.2829117)	< .001	0.161
RMSSD	3.752036 (2.251103)	10.869156 (2.546555)	2.139126 (2.226638)	< .001	0.025
pNN50	2.648027 (2.279196)	5.507546 (2.507395)	-1.775263 (2.260769)	< .001	0.032

Note: When predicting RSA, respiratory rate and BMI were entered as covariates. RMSSD and pNN50 controlled for BMI.

Table S4. Number of participants experiencing piloerection during each video.

Video	Description	Count
Woods	Neutral calibration video. A calming walk through the woods	5
The Conjuring	"The Nun" scene from The Conjuring.	6
Hill House	The final scene of "Steven sees a ghost" from Haunting of Hill House	7
Thank you, Mom	A P&G Commercial about Mothers of Olympic champions	6
10-year-old Singer	A 10-year-old singer with an operettic voice from America's Got Talent	6

Note "Count" indicates the number of participants experiencing piloerection during the video.

Biology Open • Supplementary information

Table S5. Mixed-effects regression model predicting temperature change over time between large and small piloerection.

**Term**	**Estimate**	**Std. Error**	**df**	**t**	**P**
Time	0.0005	0.0000	4,862.2304	23.4268	< .001
Size (small)	-0.0009	0.0006	633.3477	-1.5826	.114
Time x Size	-0.0002	0.0000	4,862.2304	-9.5187	< .001

Note: The significant interaction between time and size indicates that for every one-unit increase in Time, a change in the Size category from Large (the reference condition) to Small is associated with a -.0002 unit change in skin temperature. Computing marginal means and the associated Z-score reveals that, at 15 seconds after the peak of piloerection (the far right side of the graph), the two trajectories have a temperature difference of about .004 degrees Celsius (Est = .004, Z = 6.32, $p < .001$, Cohen's d = .63).
